# Hair analysis for monitoring adherence to inhaled respiratory medications: possibilities and limitations

**DOI:** 10.1007/s00228-025-03903-w

**Published:** 2025-09-09

**Authors:** Liz J. A. Cuperus, Wouter Ahmed, Johannes C. C. M. in ‘t Veen, Huib A. M. Kerstjens, Tanja R. Zijp, Jasper Stevens, A. Mireille A. Wessels, Daan J. Touw, Job F. M. van Boven

**Affiliations:** 1 Pulmonology Department, Franciscus Gasthuis & Vlietland, Rotterdam, The Netherlands; 2 Department of Pulmonary Diseases & Tuberculosis, University of Groningen, and University Medical Center Groningen, Groningen, The Netherlands; 3 Department of Respiratory Medicine, Erasmus Medical Center, Rotterdam, The Netherlands; 4 Department of Clinical Pharmacy and Pharmacology, University of Groningen, and University Medical Center Groningen, Groningen, The Netherlands; 5 Department of Anaesthesiology, University of Groningen, and University Medical Center Groningen, Groningen, The Netherlands; 6 Groningen Research Institute for Asthma and COPD (GRIAC), University of Groningen, and University Medical Center Groningen, Groningen, The Netherlands; 7 Department of Pharmaceutical Analysis, Groningen Institute of Pharmacy, University of Groningen, Groningen, The Netherlands; 8 Medication Adherence Expertise Center of the Northern Netherlands (MAECON), Groningen, The Netherlands

**Keywords:** Hair analysis, Medication adherence, Inhaled respiratory medications, Monitoring, Asthma and COPD

## Abstract

**Purpose:**

Non-adherence to inhaled medication poses a significant clinical and economic burden on patients with respiratory diseases. This narrative review provides an overview of key aspects of hair analysis, in general and specific for inhaled medications, and explores the potential of hair analysis as a novel tool to monitor adherence to inhaled medications.

**Methods:**

PubMed searches were conducted to explore four aspects: (1) mechanisms of (inhaled) drug’s systemic absorption and deposition in hair; (2) quantification of drugs in hair; (3) factors impacting (inhaled) drug hair concentrations; and (4) clinical studies assessing inhaled medication adherence through hair analysis.

**Results:**

Systemic absorption, deposition, quantification, and interpretation of drug concentrations in hair are complex phenomena and are influenced by various factors. Analysing drug concentrations in hair segments provides insights into adherence variability over up to 3 months. While studies suggest effective incorporation of several inhaled drugs into hair, inter-individual variability is influenced by external (e.g. UV-exposure), drug- (e.g. lipophilicity) and patient-specific (e.g. hair colour) factors, not just by adherence. The impact of these confounding factors on absolute hair concentrations is still unclear. Intra-individual variability unrelated to adherence appears, however, minimal.

**Conclusion:**

Although hair analysis shows promise as a novel objective bioanalytical method for assessing long-term inhaled medication adherence, until further analytical refinement, clinical validation and a clearer understanding of confounding factors, it should not be relied upon as the sole measure of adherence.

**Supplementary Information:**

The online version contains supplementary material available at 10.1007/s00228-025-03903-w.

## Introduction

A large share of patients with asthma and chronic obstructive pulmonary disease (COPD) struggle to adhere to their prescribed inhaled medication, with adherence rates ranging from 22 to 78% [1]. Non-adherence to inhaled medication is associated with sub-optimal therapeutic outcomes, decreased quality of life, and increased mortality [2–4]. Improving adherence to inhaled medications could enhance the health and overall wellbeing of patients. To achieve this goal, a prerequisite is the identification of patients with potential nonadherence. Failure to detect and optimize adherence may result in poorer treatment outcomes and/or unnecessary step-up to more expensive and/or burdensome therapies [5, 6].

A variety of methods is routinely applied to evaluate inhaled medication adherence in practice: examples include patient self-reported questionnaires, prescription records, canister weighing, or electronic monitoring [7–9]. These methods provide an indication of drug adherence, but often poorly reflect reality. Patient reports are subjective and prone to over- or underreporting adherence [10]. Prescription records and canister weighing do not translate to the true amount of (correctly) administered doses, as they provide no confirmation of (correct) intake, i.e., with adequate inhaler technique. A more direct measure of adherence can be obtained by blood sampling and subsequent measurement of drug concentrations [7, 8]. This method provides, however, only information on short-term adherence, given that the half-life of most inhaled medication typically ranges between 3 and 12 h [11, 12]. Also, this carries the risk that patients might take their medication only shortly before sampling, knowing they are being monitored (so called “white coat adherence”) [13]. An emerging method is the use of fractional exhaled nitric oxide; however, this measure is suitable for inhaled corticosteroids only and is impacted by several other factors beyond adherence [14]. Finally, digital “smart” inhalers offer an objective way to measure adherence and, depending on the type, can also assess inhaler technique [15]. However, these devices are not yet implemented in clinical practice and cannot be used to assess patients’ adherence during previous periods [4]. Given the limitations of current adherence measures, there is a need for less invasive yet objective measures, such as hair analysis [13].

Hair analysis is a minimally invasive method that could provide a retrospective record of drug exposure over a period of several months. Indeed, hair concentrations reflect historic systemic drug concentrations, with each centimetre (hair grows around 1 cm per month) corresponding to average monthly exposure. As such, hair could be particularly valuable in characterizing a patient’s historical long-term adherence [13]. In other research fields, studies have reported varying degrees of association between drug concentrations in hair and self-reported medication adherence—for instance, a weak correlation in HIV medication (*r* = 0.13) [16], but strong agreement in a study on several medications for headaches (Kappa > 0.8) [17]. So far, several studies have shown that inhaled respiratory medicines are incorporated, and subsequently detectable in the hair of patients [18–23]. Notably, the main inhaled respiratory medicines are bronchodilators and corticosteroids. Formoterol is the most frequently studied bronchodilator and was consistently quantifiable in hair across all studies [18–20, 23]. Other bronchodilators, such as salmeterol or salbutamol, were also detectable in hair [18, 21]. In contrast, studies investigating inhaled corticosteroids have shown more variable results: some were able to quantify concentrations of budesonide or fluticasone propionate [18, 23], whereas other inhaled corticosteroids, such as fluticasone furoate, were not quantifiable, likely due to its high lower limit of quantification [18].

Despite studies showing that several inhaled medications are detectable in hair and the established use of hair analysis in other research fields, primarily in forensic science, many questions remain regarding the value of hair analysis for monitoring adherence to inhaled medication. Therefore, this review aims to provide an overview of current knowledge on the key aspects of hair analysis, focusing on inhaled medications, and to review the current evidence on the use of hair analysis for monitoring adherence to inhaled medications.

### Methods

A narrative review was conducted covering four aspects of hair analysis as a tool for inhaled medication adherence monitoring: (1) mechanisms of inhaled drug’s systemic absorption and disposition in hair; (2) quantification of (inhaled) drug concentrations in hair; (3) factors impacting (inhaled) drug hair concentrations; and (4) clinical studies using hair analysis to assess inhaled medication adherence. Subparts 2 and 3 include articles on general hair analysis techniques as background, alongside articles specifically addressing inhaled drugs. The general and inhaled medication-specific information is presented in separate paragraphs. Literature searches were done in PubMed for English, full-text articles using combinations of terms “drug”, “hair”, “physiology”, “pharmacokinetic”, “extraction”, “absorption’’, “disposition’’, “quantification’’, “inhaler”, “adherence”, “monitor”, “lung”, “respiratory”, “pulmonary”, “asthma”, and “COPD”. The results were supplemented with articles known to the authors and/or identified through the snowballing method. To ensure no trials were overlooked, a more systemic search was conducted to identify the clinical studies that used hair analysis for assessment of adherence to inhaled medication. Details of this search are described in the Supplementary Material (Suppl. 1). The SANRA scale for narrative reviews was used to structure this paper [24].

## Results

### Systemic absorption of inhaled medication

**Fig. 1:**
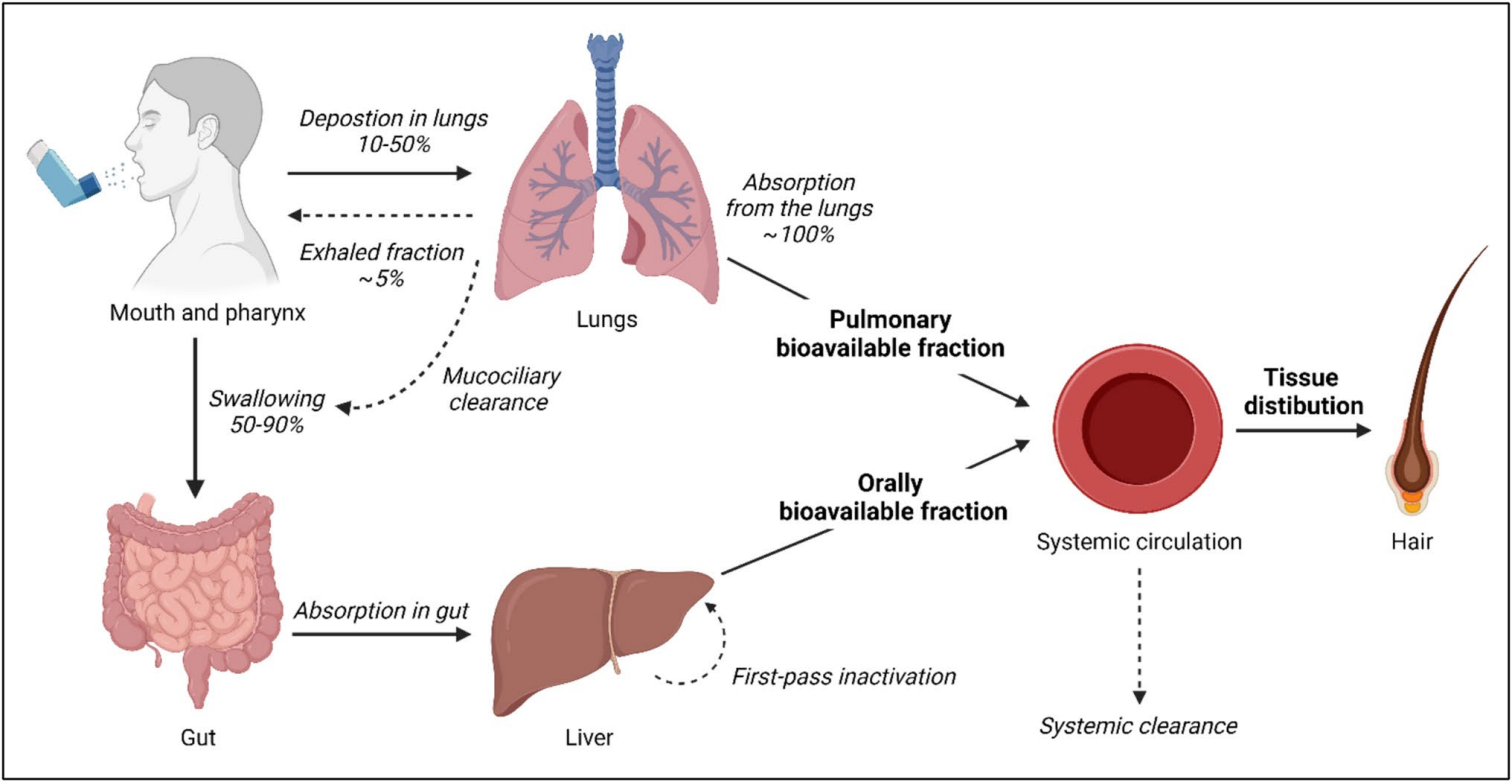
Schematic overview of the processes underlying the systemic uptake of inhaled medication. Adapted from “Relevance of pharmacokinetics and pharmacodynamics of inhaled corticosteroids to asthma” *by Derendorf, H. *et al*. The European respiratory journal 2006; 28: 1042–1050(25).* Created with BioRender.com

To detect drugs in hair, it is required that they first reach the systemic circulation and reach the smaller vessels at the root of the hair. Depending on the route of administration, drugs enter the systemic circulation through different pathways. Inhaled medications may enter the systemic circulation via the respiratory and the gastrointestinal tract (Fig. 1) [25]. Two major factors dictate the amount of inhaled medication that reaches the systemic circulation: (i) the ratio of oral to pulmonary drug deposition after actuation, and (ii) the pulmonary and oral bioavailability [25, 26].

On average, 10–50% of the dose delivered by an inhaler is deposited in the lungs, with the remainder either being exhaled (± 5%) or deposited in the oral cavity [25]. Important factors that may influence the pulmonary deposition are inhaler technique, particle size, and type of inhaler [25, 27, 28]. As a consequence of incorrect inhaler technique, a larger fraction of drug than intended can be deposited in the oral cavity and pharynx [26, 27]. A study on deposition patterns across different particle sizes showed that particle size is directly associated to the amount of lung deposition. Small (1.5 µm) particles achieve more lung deposition than medium (3 µm) and large (6 µm) particles: 56.3%, 51.0%, and 46.0% of the administered dose, respectively [28]. Consequently, deposition patterns of different types of inhalers used in respiratory disease management vary greatly [25, 28, 29]. A systematic literature review showed lung deposition rates of 8–53% for pressurized metered-dose inhalers (pMDIs), 7–69% for dry powder inhalers (DPIs), and 39–67% for soft mist inhalers (SMI) [30]. Furthermore, there is significant interindividual variability in drug deposition ratios with reported coefficients of variation of 8.2% and 61.2% in asthmatic patients using a pMDI or a DPI, respectively [31]. Pulmonary bioavailability is usually similar to the deposition rates given there is no first-pass metabolism by the liver after absorption from the lungs. Note that a small fraction of the lung-deposited dose may never reach the systemic circulation as a consequence of metabolism in lung epithelium or mucociliary transport to the pharynx [32].

A substantial part of the delivered dose is deposited in the gut (50–90%). Oral bioavailability ranges from negligible to relevant values for both inhaled corticosteroids (e.g. < 1% for ciclesonide and fluticasone propionate to 26% for beclomethasone monopropionate) as well as for long-acting bronchodilators (e.g. < 1% umeclidinium to 17% for formoterol) [11, 25, 33, 34].

### Inhaled drug disposition in hair

From the systemic circulation, a small part of the inhaled drug is transferred to hair tissue. However, the general mechanism of drug disposition in hair is poorly understood. The prevailing theory suggests that multiple pathways could contribute to this process [35–37].

#### Incorporation from systemic circulation

The most prominently described route involves passive drug diffusion from the capillaries into the growing hair cells at the base of the hair follicle (i.e. the hair matrix) [35–37]. Drug-containing hair cells then slowly ascend to the hair shaft, where they elongate and increase in volume. Finally, keratinization occurs and drug becomes embedded in the non-living hair fibre [36–38]. Over time, the distance between hair-bound drug and the base of the follicle will increase as hair grows. The Society of Hair Testing (SoHT) reports that the generally accepted rate of (scalp) hair growth is 1 cm per month. A literature review reported similar hair growth rates (mean hair growth rate ± SD: 1.06 ± 0.06 cm/month) [39–42]. Variations in drug incorporation into hair can be partially attributed to differences in physicochemical properties such as acidity (pKa) and lipophilicity (logP), which influence diffusion efficiency. In particular, lipophilic and basic drugs are typically incorporated into hair in greater amounts. Basic drugs may be incorporated up to 40 times more than structurally similar neutral or acidic compounds [35, 43–46].

#### Sebum or sweat

Drug deposition in hair also occurs through sebum, a fatty substance secreted by the sebaceous glands that coats the hair and plays an important role in hair lubrication [47–49]. Via contact between drug-containing sebum (e.g. through passive diffusion into the sebaceous gland) and hair, drugs bind superficially to the external part of the hair shaft [35–37]. In addition, sebum-mediated hair binding may also result in some internal drug incorporation due to the porous nature of hair [36, 50]. Similarly, it has been shown that drugs are detectable in sweat [35–37, 51–53]. Two studies have demonstrated that drug-free hair can acquire measurable amounts of drugs through sweat-to-hair contact when handled by someone under the influence of drugs of abuse [36, 54].

#### Other sources

Environmental factors may also play a role in the deposition of drug into hair, most notably through contact with drug-containing air or water [35–37]. For example, passive external exposure to marijuana smoke or vaporized cocaine can lead to the contamination of hair through airborne deposition of contaminants [55, 56]. Theoretically, this may also apply to sprayed aerosols from inhalers, although no studies have been found addressing this. Lastly, it has been hypothesized that drug deposition from skin into hair could occur as a result of the transport of lipophilic drugs through skin cells and layers [35–37].

### Quantification of drug concentrations in hair

**Fig. 2:**
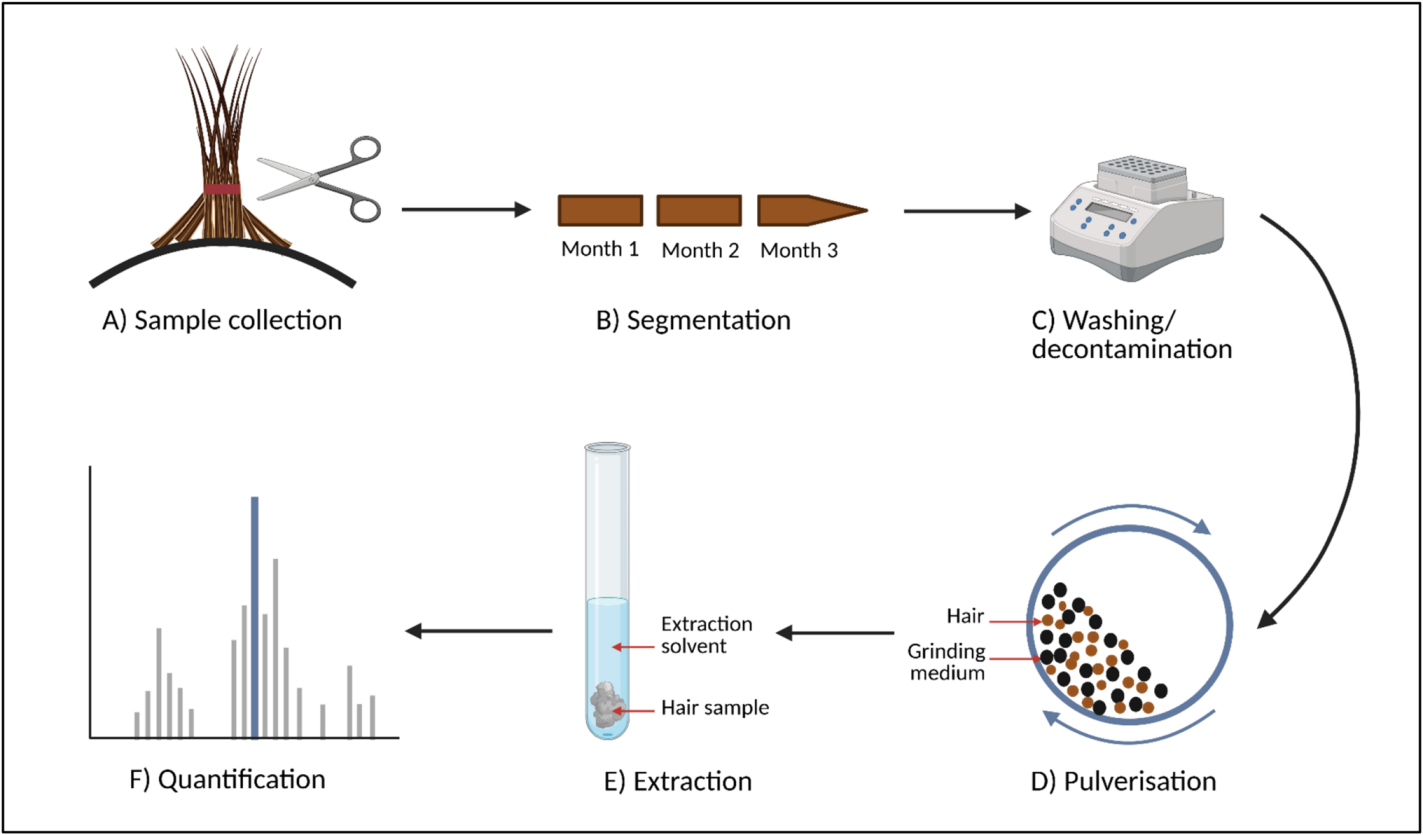
Schematic overview of different steps in the hair analysis process: **a** sample collection, **b** segmentation of a hair strand into monthly segments, **c** decontamination using different wash solutions, **d** pulverization of hair sample, **e** extraction of drug embedded in the pulverized hair, and **f** quantification of drug concentrations in hair. Created in https://BioRender.com

The quantification of inhaled drugs follows a process similar to that used for quantifying other, non-inhaled drugs. To actually detect and quantify drug concentrations, three steps are relevant: (i) sampling, (ii) preparation, and (iii) testing (Fig. 2) [40, 42].

#### Sampling

The SoHT recommends that hair is sampled from the posterior vertex region of the scalp (Fig. 2A). This area shows the smallest variation in hair growth rates, has the highest percentage of hairs that are growing at one time, and the fastest growth rate [40–42]. Other sampling sites may be chosen if considered more appropriate (e.g. in case of baldness). Samples should ideally be cut as close to the scalp as possible to avoid missing a period of drug exposure [39, 42, 58]. Still, even when applying this sampling method, an average of 0.8 cm of hair can remain on the scalp [39]. Furthermore, newly formed hair lies approximately 0.4 cm below the scalp surface and, based on the average monthly rate of hair growth of 1 cm, it will take approximately 2 weeks to reach the skin surface [39, 59]. This gap of roughly one month of drug exposure when cutting hair should be taken into account prior to sampling. Hence, it is recommended to wait 8 weeks after the last exposure of interest before sampling [39]. Hair samples should be collected in a contamination-free area, with approximately half the thickness of a pencil providing sufficient hair for subsequent testing procedures. Retrieved samples should then be stored in a dark and dry place at room temperature, since refrigeration or freezing might introduce swelling that could result in the loss of drug [40, 42].

#### Preparation

##### Segmentation

As an optional step in the preparation phase, hair could be cut into different segments (Fig. 2B), allowing comparison of medication use over different time periods [42]. Therefore, segmentation could be of particular use in assessing intraindividual variability in medication adherence. Although it may seem straightforward, dividing hair into “monthly” or “quarterly” (1 cm and 3 cm, respectively) segments requires careful consideration of interindividual variability in hair growth rates. If hair is cut into 1 cm segments, estimates of a patient’s true monthly adherence levels may be inaccurate if their hair growth rate significantly deviates from the average.

##### Washing

As a next step in the preparation phase, washing is recommended to remove hair care products, sweat, sebum, skin cells, residual drug or anything else from the external part of the hair (Fig. 2C) [42]. Many different approaches to hair washing have been described in the literature [60, 61]. The effect of washing has been up for debate, as studies continue to show measurable amounts of drug in externally contaminated hair, even after the most thorough washes [36, 42, 60, 62, 63]. Conversely, thorough washing also carries the risk of removing the drug from the porous interior of the hair through hair swelling [58]. This same mechanism is also hypothesized to be responsible for the transfer of externally bound drug to the (internal) hair cortex and medulla described in literature [60, 64, 65]. The SoHT recommends that any wash procedure is laboratory validated, prior to being used in practice [40, 42].

In previous studies investigating inhaled medications, hair decontamination was done either by washing twice with 5 ml methylene chloride for 2 min, or with hot distilled water for 2 min [19–23].

Our own research group is washing the samples twice by 3 ml dichloromethane for 10 min in an ongoing trial investigating the correlation between formoterol concentrations in hair and digital inhaler data [66].

#### Testing

##### Extraction

The extraction procedure aims to release drugs that are embedded in the largely inaccessible internal hair matrix into an extraction solvent for analysis [67]. A crucial first step in the extraction process is pulverization (i.e. the milling of hair using small metal balls) (Fig. 2D). Pulverization increases the hair’s surface area, resulting in greater drug extraction [68]. This could increase the extraction of drugs from hair to 1.8-fold [20]. A concern regarding the use of pulverization is the potential heating of the sample, which may lead to the degradation of thermosensitive medications. Therefore, cooling of hair prior to and during the process of milling is recommended by some mill manufacturers and is often applied in practice [68]. As an alternative to pulverization, the hair can also be digested or cut into small pieces to homogenize the sample [42]. After pulverization, drugs can be extracted from the pulverized hair through a range of methods (Fig. 2E). Most importantly, a combination of enzymatic digestion, digestion with an aqueous acid buffer solution, organic solvent extraction, liquid–liquid extraction, and solid-phase extraction (SPE) methods are commonly used and have been described in great detail elsewhere [61, 67]. The optimal extraction procedure depends on the chemical properties of the analyte and will consequently differ across medications [61, 67].

For inhaled medications, a variety of extraction methods have been used, differing in specific procedures such as pulverization time, extraction fluids, and other processing steps [18–22]. For instance, both Salvator et al. and our research team used extraction methods based on suggestions previously made by Lamy et al. [19, 20, 23]. We used 1.0 ml methanol as the extraction fluid, pulverization with small metal balls for 60 min, centrifugation for 5 min, and transferring the supernatant into Whatman filter vials [19]. In contrast, Cirimele et al. washed twice in 5 ml methylene chloride for 2 min each and then pulverized in a ball mill. Regardless of the method used, it is impossible to determine whether all the medication has been extracted, as measuring the residue left behind in the hair is not possible.

##### Quantification

After extraction of the analyte from hair, the compounds present in the obtained extract are separated and the drug of interest is quantified (Fig. 2F) [42, 61]. For hair analysis of drugs in general, analytical methods that are routinely used for the separation of compounds are liquid- and gas chromatography (LC and GC) [61]. Mass spectrometry (MS) is the preferred method for detecting and quantifying drugs in hair, as it is the most sensitive tool available for detecting the typically low drug concentrations (in the range of pg/mg hair) found in hair [42, 61].

Specifically for quantification of inhaled drugs, a LC–MS method has been published that allows for the quantification of a range of inhaled corticosteroids, β_2_-adrenoreceptor agonists, and anticholinergics in hair [20]. This method was shown to be highly sensitive, suitable for the analysis of samples originating from patients, and has been validated according to the European Medicines Agency and Food and Drug Administration guidelines [69, 70].

##### Bioanalytical validation

The bioanalytical validation procedure in general involves assessing the method’s selectivity, lower limit of quantification (LLOQ), calibration range, accuracy, precision, matrix effects, and the stability of the analyte(s) [70, 71]. Validation of a bioanalytical method developed for the quantification of drug in hair is, i.e., compared to plasma, often more difficult for two reasons. First, spiking human hair samples with the drug of interest cannot replicate authentic hair samples from patients [42, 65, 72]. Second, no certified reference materials for hair containing inhaled medication are available [20]. The SoHT suggests using hair samples from individuals whose exact medication usage is known to ensure the performance of a bioanalytical method [42, 72]. Alternatively, researchers could opt for the approach presented by Lamy and colleagues [20]. Here, “blank” hair samples were spiked with working solution followed by the extraction process. This approach was modified by our research team by adding the extraction solvent to the blank hair samples and pulverizing the samples in a ball mill. After centrifugation, the obtained extract was then spiked prior to chromatographic analysis (details can be provided upon correspondence with authors) [19].

### Clinical validation

Clinical validation should be performed before the novel bioanalytical method is implemented in clinical practice. Currently, no guidelines exist for evaluating the clinical applicability of hair as a medication adherence monitoring tool. Several recommendations have been made by authors of a scoping review on bioanalytical methods for drug measurements [13]. Correlations between drug concentrations in hair and established adherence monitoring methods related to long-term exposure (e.g. dose counting) should be demonstrated. In addition, clinical validation may also involve the correlation of inhaled drug concentrations in hair with disease outcomes. Notably, an ongoing trial is exploring the potential correlation between formoterol concentrations in hair, electronic monitoring via smart inhalers, and adherence levels reported through the Test of Adherence to Inhalers (TAI) questionnaire [66].

### Factors impacting drug concentrations in hair in general

**Fig. 3:**
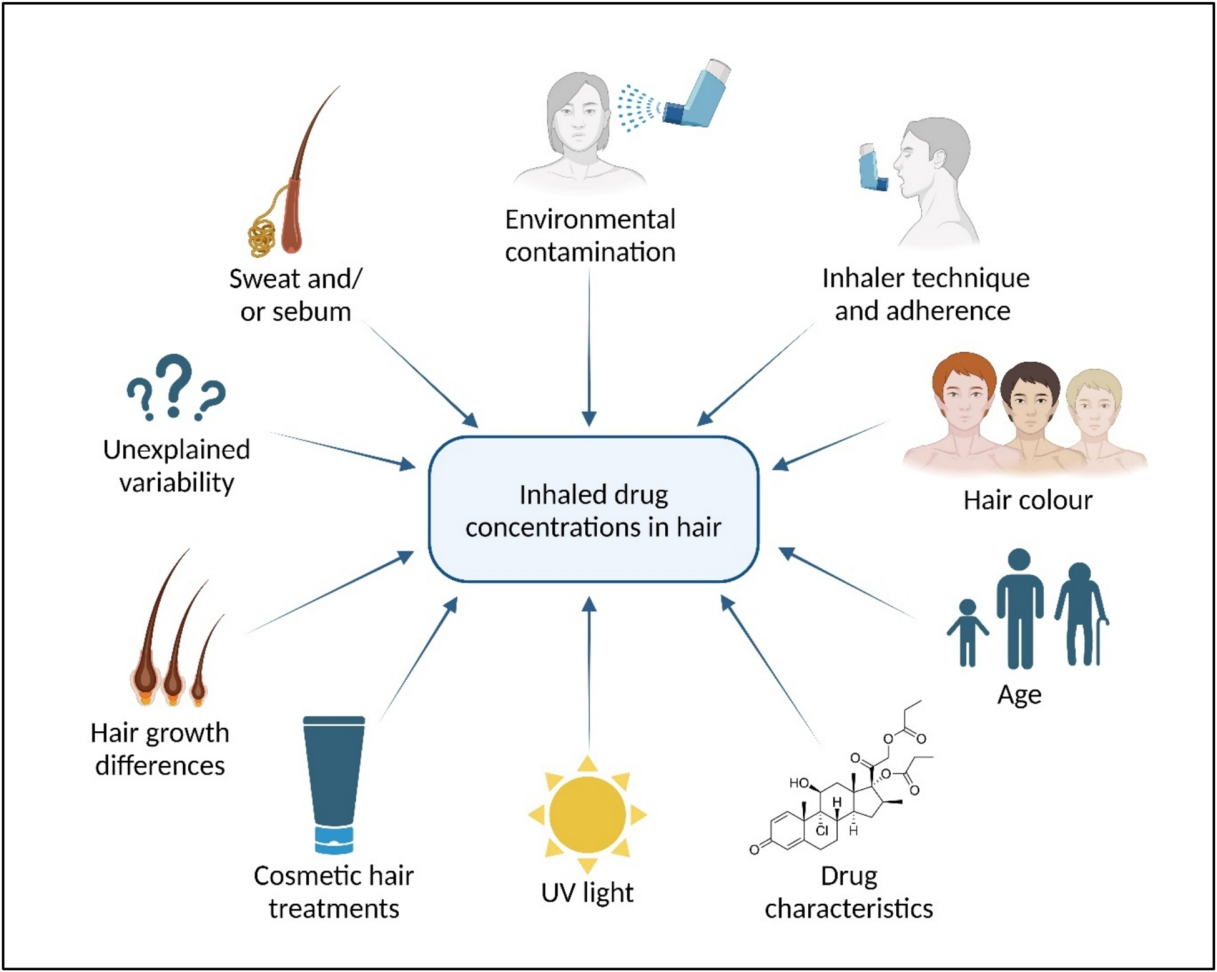
Factors influencing the inhaled drug concentrations in hair. Created in https://BioRender.com

The correlation between measured drug concentrations in hair and the corresponding actual intake (i.e. medication adherence) is pivotal for clinical interpretation, but is not straightforward [57, 61, 73] and impacted by several factors as has been shown for multiple (non-inhaled) medications [74–78]. Notably, these factors affect both the inter- and intraindividual variability of hair drug concentrations (Fig. 3). Several factors affect drug concentrations in hair, regardless of the route of administration. These include hair colour, the drug’s chemical properties, exposure to UV light, and cosmetic hair treatments.

#### Hair colour

Apart from hair protein- and lipid-structures, melanin has been described as one of the primary binding sites of drugs deposited in hair [37]. Therefore, hair colour is an important determinant of the amount of drug that can be deposited in hair. Numerous studies have shown that a range of drugs administered at similar dosages deposit to greater amounts in darker, and thus hair with more melanin, compared to lighter hair [81–86]. In addition, an age effect of drug-hair binding has been reported in literature, likely as a result of the decreasing melanin content in hair with age (i.e. hair greying) [50, 84, 87]. To prevent incorrect conclusions as a result of differences in melanin content, Rollins et al. propose to “normalize” measured concentrations in hair by expressing them as a function of melanin concentration rather than hair weight. Using codeine as an example, the authors show that concentrations found in hair of different colours are more comparable after normalization [81]. Generally, the need for a “melanin correction” should be evaluated on a drug-to-drug basis, as the so-called colour bias in hair binding has not been established for all drugs [88, 89].

The potential impact of hair colour on inhaled medications has not been widely studied; however, the binding of formoterol to hair has been shown to be melanin-dependent in one study [18].

#### Drug characteristics

The properties of a drug itself are also relevant for incorporation in hair. Properties such as high lipophilicity, absence of an acidic function, and long N-alkyl chains and N-benzene rings have all been described as favourable [35, 90]. The drugs’ physicochemical properties may also clarify why certain compounds are more easily detected in hair than others. As described earlier in this article, basic drugs are incorporated into hair in up to 40-fold larger quantities than structurally similar neutral or acidic compounds. Furthermore, other factors such as half-life time and the LLOQ (and the sensitivity of the used equipment) may also influence the detectability of the drug in hair.

Lipophilicity, acidity, and half-life of several inhaled medications are displayed in Table 1.

#### Other external influences

The external position of hair on the human body not only puts it at risk for external contamination with the drug of interest, but also to various chemical and non-chemical influences that have the potential to affect drugs deposited in hair. These effects accumulate over time, so after a few months of exposure, they become increasingly significant. As a result, only the first 3 cm of hair from the scalp (equivalent to about 3 months) are typically appropriate for analysis [42].

##### UV light

Degradation of drug concentrations in hair has been observed for several drugs, such as ketamine and amphetamines [93]. The ideal site of hair sampling, i.e. the scalp, is often in direct contact with sunlight for extended periods of time throughout the day. As a result, UV light induced photodegradation of drugs in hair may occur which could result in an underestimation of the level of adherence. Alternatively, the photodegradation of lipids, proteins, or melanin present in hair might also act as a mechanism of hair-bound drug loss [94].

The impact of UV light on the concentrations of inhaled medications in hair has not been studied. However, exposure to UV light has been shown to result in the degradation of commonly used inhaled medications, salbutamol and budesonide, in a gel or in environmental water [91, 92]. Furthermore, the results of studies with non-inhaled drugs may suggest that UV exposure may also degrade inhaled medications in hair.

### Cosmetic hair treatments

Given that cosmetic hair treatments can also impact drug concentrations in hair, it is essential that use of any cosmetic hair treatments is recorded and taken into consideration when interpreting hair-drug concentrations [42]. Apart from hair damage, the conversion or degradation of drug present in hair as a result of cosmetic hair treatments has been proposed as a mechanism of drug loss [50, 95]. Bleaching has been shown to reduce methamphetamine concentrations in hair, with the extent of reduction depending on the severity of the bleaching. Mild bleaching resulted in an average reduction of 13.8% (range: 1.5%–29.9%), moderate bleaching caused a 37.9% reduction (range: 10.0%–59.6%), and severe bleaching led to a 69.3% reduction (range: 44.3%–91.4%) [96]. Perming hair results in comparable reductions in drug concentrations, ranging from 28.1% to 94.7%. Dyeing hair can also cause drug loss, though to a lesser extent than bleaching and perming. Depending on the type of dye used, the average reduction in drug concentration ranges from 0.1% to 24.0% [96, 97]. Hair care products such as conditioners and shampoos can either increase or decrease hair-drug concentrations. For example, one study found that washing reduced tetrahydrocannabinol (THC) concentrations in hair by an average of 52%–65%, depending on the shampoo type [98]. Conversely, Kidwell et al. demonstrated that hair care products containing hydrophilic components increased the uptake of drugs like cocaine and methamphetamine compared to oil-based products. This effect occurs because water exposure causes hair to swell, making it easier for drugs to be incorporated [99]. Future research will have to elucidate how different cosmetic hair treatments and hair care products affect hair binding of various inhaled medications.

### Factors impacting inhaled drug concentrations specifically

Some factors are specific to inhaled drugs, which may lead to different drug concentrations than merely expected via pulmonary drug deposition after actuation [32, 35–37]. As discussed earlier in the section “Systemic absorption of inhaled medication”, contamination caused by aerosol particles depositing on the hair or being exhaled into the air could theoretically influence the concentration in hair. More importantly, inhalation technique may have a significant impact on the measured drug concentrations and, consequently, on the interpretation of the results.

#### Inhaler technique

An observational study in a real-life setting showed that 49–76% of patients made at least one error while using their inhaler [79]. As a result of incorrect inhaler technique, the ratio of oral to pulmonary drug deposition may become larger [80]. The exact impact of inhaler technique on systemic exposure and consequently on hair concentrations depends on the inhaled drug’s specific actual oral and pulmonary bioavailability [25]. For instance, fluticasone propionate has negligible oral bioavailability (< 1%). When inhalation technique is poor, a smaller portion of the drug dose reaches the lungs and systemic circulation, leading to lower concentrations in hair. However, the concentration in hair can be fully attributed to the fraction of the drug that entered through the intended route (i.e., the lungs), making the results easier to interpret. In contrast, interpreting drug concentrations in hair from inhaled medications with higher oral bioavailability, such as beclomethasone dipropionate (15%) or formoterol (17%), is more challenging. These drugs are more affected by incorrect inhalation technique, as this could result in elevated systemic circulation and consequently higher hair concentrations without sufficient lung deposition [26].

### Clinical studies using hair analysis to assess inhaled medication adherence

Hair analysis has been used in several clinical studies that investigated adherence to inhaled medication (Table 1). The studies varied in study population, number of participants and drugs tested. Most samples collected were of sufficient quality for analysis (76%–100%), meaning there was an adequate amount of hair, the samples were properly cut and stored, and the scalp end was clearly identifiable. However, the studies differed in the proportion of samples in which the drug could be quantified (ranging from 40 to 100%). Furthermore, some drugs could not be detected, e.g. beclomethasone in the study of Hassall et al. [18]. Formoterol was the only drug that was tested in all studies. A wide range of formoterol concentrations in hair has been observed with the lowest value of 0.6 pg/mg reported by Salvator et al. and the highest value of 500 ng/g (pg/mg) reported by Hassall et al. [18, 23]. Besides measuring the drug concentrations, the studies also investigated the correlation with adherence. Dierick et al. compared the drug hair concentrations with digital spacer usage data; however, the small patient sample size (*n* = 6) prevented them from drawing any conclusions. In the paper of Hassall et al., a dose–response relationship between the formoterol daily dose and its concentration in hair was observed. Furthermore, relationships between formoterol concentration in hair and self-reported adherence and hair colour (higher formoterol concentrations in darker hair) were suggested. However, it should be noted that this was investigated in a limited number of patients and no statistical tests were conducted [18]. Salvator et al. examined the correlation between drug concentration in hair and both self-reported drug dose (Pearson’s coefficient: 0.42, *p* = 0.08 for budesonide (BUD); 0.24, *p* = 0.44 for formoterol (FM)) and prescribed dose (Pearson’s coefficient: 0.29, *p* = 0.25 for BUD; 0.17, *p* = 0.57 for FM). They also evaluated inter- and intrapatient variability for budesonide (*n* = 18) and formoterol (*n* = 13). Intrapatient variability, measured over the 4-month study period, was lower in budesonide (20.8% [13.2–31.4]) compared to formoterol (39.9% [28.6–64.7]). Inter-patient variability was high for both budesonide (109%) and formoterol (118%), even after normalization for the received dose (respectively 69.7% and 108%) [23].

**Table 1:** Overview of clinical studies using hair analysis to assess inhaled medication adherence

Reference	Number of patients	Disease	Drug(s) tested	Samples suitable for analysis, *number (%)*	Drug quantifiable in hair, *number (% of samples analysed)*	Drug concentration in hair,* mean/median [range] in pg/mg or ng/g*^*1*^	LLOQ, *in ng/g*	Drug characteristics *Source: PubChem, Drugbank Online and Derendorf *et al [25, 33, 34]				
								Drug	Oral bioavailability (%)	Half-life, *in hours*	Hydro-/lipophilic^3^ (logP)	Acidic/basic^3^ (pKa)
Dierick et al. [19]	6	COPD	FM	6 (100)	6 (100)	Pre-intervention: 1.5 [1.1–1.9] pg/mg; Post-intervention: 1.2 [1.0–1.4] pg/mg		FM	17	7–10	Lipo (2.2)	Basic (7.9–9.2)
Hassall et al. [18]	200	Asthma and/or COPD	FF, FP, FM, SAL, TIO, VI, UMEC	152 (76)	61 (40)	^2^FF *not shown*	160	FF	1.3	24	Lipo (3.73–4.13)	Basic (13.61)
						FP [18–1000] ng/g;	80	FP	< 1	7.8 (iv)	Lipo (2.78)	Basic (13.61)
						FM [0.8–500] ng/g;	0.8	FM	17	7–10	Lipo (2.2)	Basic (7.9–9.2)
						SAL [6–25] ng/g;	4	SAL	?	5.5	Lipo (4.2)	Basic (11.2)
						TIO *not shown*	0.4	TIO	2–3	24–44	Hydro (− 1.8)	Basic (10.35)
						VI [0–1.4] ng/g	2	VI	< 2	11	Lipo (3.39–3.6)	Basic (9.4–10.12)
						UMEC *not shown*	8	UMEC	< 1	11	Neutral (0.68–2.88)	Basic (13.04)
						BDP *not shown*	*not detectable*	BDP	15	8.8	Lipo (3.49)	Basic (13.85)
Salvator et al.[23]	21	Asthma	BUD, FM	21 (100)	BUD: 18 (85.7)	BUD: 6.3 [3.6–8.5] pg/mg		BUD	11	2–3.6	Lipo (1.91)	Basic (13.75)
					FM: 13 (61.9)	FM: 0.9 [0.6–3.9] pg/mg		FM	17	7–10	Lipo (2.2)	Basic (7.9–9.2)

Abbreviations: Formoterol (FM), Fluticasone furoate (FF), Fluticasone propionate (FP), Salmeterol (SAL); Tiotropium (TIO), Vilanterol (VI); Umeclidinium (UMEC), Budesonide (BUD), Beclomethasone dipropionate (BDP), Lower Limit of Quantification (LLOQ).

^1^Concentrations are reported in the units used in the original publications (pg/mg or ng/g), which are equivalent and allow for cross-study comparison.

^2^Ranges of drug concentrations measured in hair are estimated from Fig. 2 in the paper of Hassall et al. [18]. Mean or median concentrations were not provided.

^3^Lipophilic (Lipo) and basic (Basic) drugs are better incorporated in hair compared to hydrophilic (Hydro) and acidic (Acidic) drugs.

The left side of the table displays the results of the studies; the right side shows the characteristics of specific drugs that may help explain differences in measured concentrations in hair

## Discussion

This narrative review highlighted aspects regarding the usefulness of hair analysis to monitor inhaled medication adherence. On the one hand, hair analysis has several advantages. Sample collection is non-invasive, retrieved samples are stable, and their analysis provides the ability to retrospectively evaluate medication use over the long term. These same advantages have also led to the exploration of hair analysis to assess long-term cortisol concentrations as a non-invasive tool for measuring adrenal function in children with asthma [100].

On the other hand, many questions remain to be addressed. Most importantly, large interindividual variability in inhaled drug concentrations in hair may occur through physiological (e.g. hair colour or growth rates) and external factors (e.g. external drug binding or hair product or UV light induced drug loss) and this variation needs to be better quantified and understood. Furthermore, differences in the detectability of various inhaled drugs in hair have been observed, likely due to their distinct characteristics. For instance, Hassall et al. detected fluticasone in hair, but not beclomethasone [18]. Although these drugs have similar acidity (pKa) and lipophilicity (logP), fluticasone has a much longer half-life than beclomethasone, whereas beclomethasone has higher oral availability. One could hypothesize that the relatively longer exposure time seems more important for the incorporation from the systemic circulation in the hair vessel, resulting in higher concentrations. However, also differences in molecular structures may be responsible. Notably, it is currently difficult to directly link measured inhaled drug concentrations in hair to an individual’s level of adherence. This seems especially true for patients who have an incorrect inhaler technique, as poor technique results in relatively more orally available medication. This could lead to either elevated drug concentrations in hair without sufficient lung deposition for drugs with significant oral bioavailability or lower concentrations in hair for drugs with negligible oral bioavailability. While a dose–response relationship between hair concentrations and self-reported adherence has been observed for formoterol [18], for many other inhaled drugs, this relationship remains unclear.

In our view, unlike its use in forensic, legal, or employment testing, hair analysis is not yet ready for use in clinical practice for monitoring of adherence to inhaled medications [13, 57]. At best, hair analysis can be used to confirm intake (binary: yes/no) over the last three months in some inhaled medications, such as formoterol and budesonide. This may make it potentially interesting as a “point-of-care” test to justify treatment escalation, e.g. to biologic therapy, as this often occurs in patients who are actually nonadherent to their inhaled medicines [101]. However, not all inhaled medications were detectable in hair, making such a confirmatory test of drug presence impossible for these compounds. For example, beclomethasone could not be measured, likely due to its high lower limit of quantification [18]. Our research team has faced the same limitation and is currently unable to detect beclomethasone in an ongoing trial [66]. With future advancements in analytical sensitivity, detection of drugs with higher LLOQs may become feasible. Moreover, poor understanding of causes and impact of interindividual variability makes hair analysis currently less appropriate for quantifying adherence. Hair analysis could also serve as a complementary tool to established methods for assessing adherence to inhaled medications; for instance, alongside prescription records or electronic monitoring. Following the hair analysis, adherence could be further evaluated using tools like the Test of the Adherence to Inhalers (TAI) questionnaire to explore potential reasons for nonadherence and subsequently optimize it using tailored interventions [102]. Furthermore, hair analysis may be used to evaluate intraindividual variability in adherence, i.e. in a single patient over time, to assess the effect of interventions to improve adherence. Note however that some (not adherence related) intraindividual variability in drug concentrations could exist, though to a lesser degree than interindividual variability.

In conclusion, while hair analysis currently has limited value as a standalone method for measuring adherence to inhaled medications, it may serve as a complementary tool alongside other adherence assessment methods. The limited evidence regarding inhaled medications in hair has likely constrained our ability to fully evaluate its potential and practical benefits. Future studies should involve larger cohorts in real-world settings and aim to further validate this technique for inhaled medications and to assess the impact of external and physiological factors on inhaled drug concentrations in hair. A deeper understanding and quantification of these sources of variability could enhance the use of hair analysis for monitoring adherence to inhaled medications.

## Supplementary Information

Below is the link to the electronic supplementary material.

Supplementary file1 (DOCX 63 KB)

## Data Availability

No datasets were generated or analysed during the current study.
