# Supplementary material for: Hair analysis for monitoring adherence to inhaled respiratory medications: possibilities and limitations
Source: Eur J Clin Pharmacol. 2025 Sep 9;81(12):1745–58. doi: 10.1007/s00228-025-03903-w (PMC12680825; doi:10.1007/s00228-025-03903-w)

## Supplementary material

**Supplement 1. Details literature search to identify clinical studies that used hair analysis to assess inhaled medication adherence**

*Inclusion criteria:*

- Original papers
- Population = patients with asthma and/or COPD
- Outcome = adherence to inhalation medication
- Measurement method of adherence= hair analysis of drug concentrations

*Search strategy in PubMed (search date: 04-09-2024)*

("Lung Diseases, Obstructive"[Mesh] OR obstructive-lung-disease*[tiab] OR COPD[tiab] OR Asthma*[tiab] OR Obstructive-pulmonary-disease*[tiab] OR COAD[tiab] OR obstructive-airway-disease*[tiab] OR chronic-airflow-obstruction*[tiab] OR chronic-airway-obstruction*[tiab] OR bronchitis*[tiab] OR bronchiolit*[tiab] OR emphysem*[tiab] OR bronchopneumoni*[tiab] OR broncho-pneumoni*[tiab] OR cryptogenic-organizing-pneumoni*[tiab] OR BOOP[tiab]) AND ("Hair Analysis"[Mesh] OR "Hair/chemistry"[Mesh] OR (hair*[tiab] AND analy*[tiab])) AND ("Treatment Adherence and Compliance"[Mesh] OR adher*[tiab] OR nonadher*[tiab] OR comply*[tiab] OR noncomply*[tiab] OR complian*[tiab] OR noncomplian*[tiab])


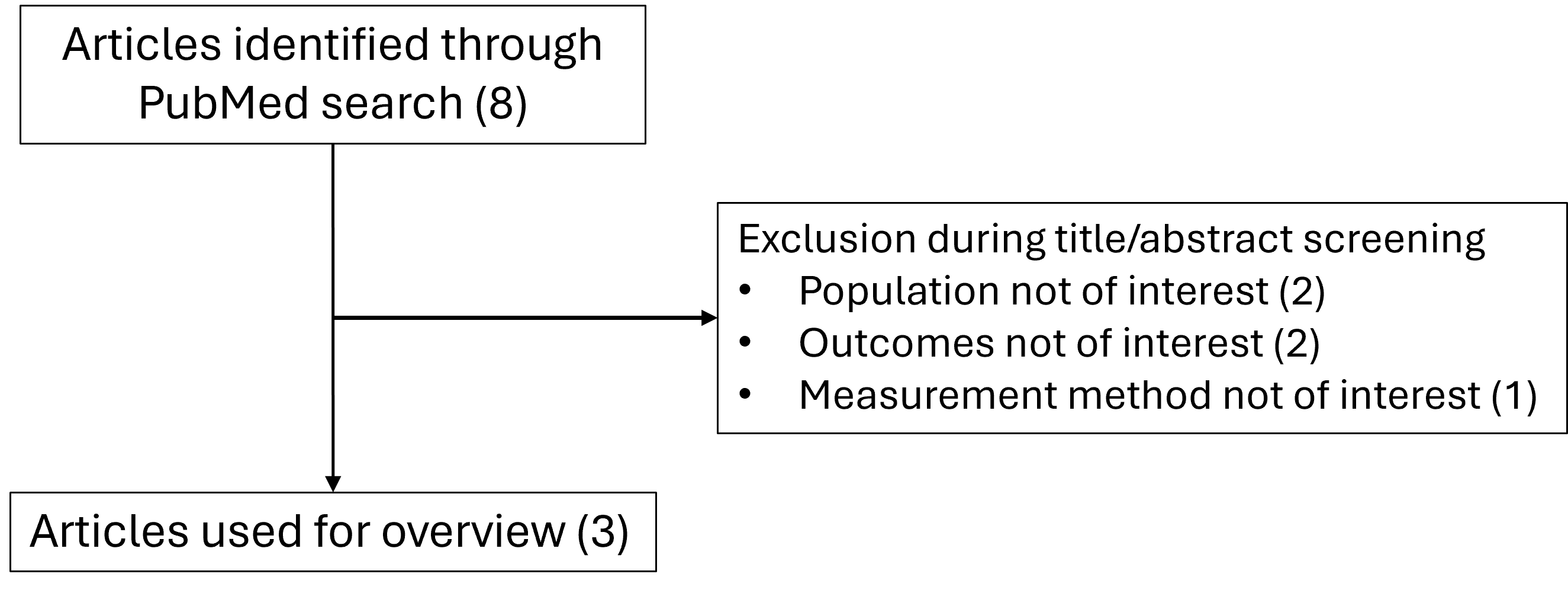

Supplement: Supplementary file 1 — Supplementary file1 (DOCX 63 KB) [file 228_2025_3903_MOESM1_ESM.docx]
